# Least squares reverse time migration imaging with illumination preconditioned based on improved PRP conjugate gradients

**DOI:** 10.1038/s41598-023-40578-8

**Published:** 2023-08-21

**Authors:** Xiaodan Zhang, Rui Li, Lin Cui, Dongxiao Liu, Guizhong Liu, Zhiyu Zhang

**Affiliations:** 1 School of Electronic Information, Xi’an Polytechnic University, Xi’an, 710060 China; 2 School of Information and Communications Engineering, Xi’an JiaoTong University, Xi’an, China; 3 School of Automation and Information Engineering, Xi’an University of Technology, Xi’an, China

**Keywords:** Solid Earth sciences, Geophysics

## Abstract

Least squares reverse time migration (LSRTM) imaging is the one of the most accurate methods for migration imaging at present, and Polak–Ribiere–Polyak conjugate gradient (PRPCG) for LSRTM has the good numerical performance but weak convergence, so we construct an optimization factor to improve the iteration direction of the gradient, which can automatically generate a sufficient descent direction. The improved PRPCG (IPRPCG) can reduce the data residual values and speed up the iteration. And the illumination preconditioned (IP) operator is employed to IPRPCG-LSRTM which solves the problem of low resolution due to the insufficient iterative gradient information. In this paper, the experiments show that the imaging results of the proposed method (IPRPCG-IP-LSRTM) is improved greatly in detail characterization and events continuity, the iterative curve converged faster significantly, and the normalized data residual was reduced by 6.55% on average, which improved the accuracy of migration imaging effectively.

## Introduction

With the development of oil and gas exploration, it poses greater challenges to traditional migration imaging methods for underground structure imaging^1,2^. In order to improve imaging accuracy and amplitude fidelity, researchers proposed least squares reverse time migration (LSRTM)^3,4^. LSRTM often uses Conjugate Gradient (CG) algorithm to find the optimal solution. The classical CG methods include the Fletcher–Reeves (FR) , Polak-Ribiere-Polyak (PRP) , Hestenes Stiefel (HS), and Conjugate Descent (CD) method. Their different iteration directions and step sizes will show different convergence rates^5,6^. Therefor, it is an important topic in the application research of LSRTM to research the new algorithm of iteration directions to achieve global convergence^7,8^. For the problem of insufficient illumination in deep subsurface imaging^9–11^, which leads to insufficient iterative gradient information resulting in low imaging resolution and slow convergence of iterative curves, illumination preprocessing has been developed and has attracted extensive research in academia^12–14^.

As for the development of conjugate gradient algorithm and illumination preprocessing, Liu et al.^15^ performed time-domain full waveform inversion for eight versions of the CG method, and numerical experiments showed that the PRP conjugate gradient (PRPCG) method is more efficient. Kim et al.^16^ proposed direction-oriented wavefield imaging, which compensates for possible illumination effects during acquisition and can produce high-fidelity depth profiles and correct imaging of complex wavepaths. Yu et al.^17^ developed a 3D nonlinear conjugate gradient traveltime inversion method using the PRP conjugate gradient method for solving constrained damping least squares problems, and model tests showed that the method can substantially improve the inversion resolution. Chen et al.^18^ proposed the use of a wave illumination compensation method for the possible existence of offset shadows in seismic wave illumination, which can eliminate the offset imaging shadows and improve the computational efficiency. Wu et al.^19^ introduced the reweighted regularized conjugate gradient method of PRP formula to constrain the inversion for the "skin effect" problem in gravity data inversion, which improved the computational efficiency and accuracy of the inversion. Sun et al.^20^ developed a stabilization compensation operator that attenuates poor illumination of subsurface structures and does not amplify high frequency noise in the data. Hu et al.^21^ derived the gradient of the target generalized function on the model parameters from the Love wave fluctuation equation combined with the PRP conjugate gradient algorithm, and model tests verified that the method can improve the computational efficiency. Du et al.^22^ used log-absolute error functions for AVO inversion and used a new spectral PRP conjugate gradient method in iterations to solve large-scale optimization problems, and then they combined a smooth nonconvex regularization method with adaptive individual weight gain and used a PRP conjugate gradient method to minimize the objective function^23^, recently they used a smooth L1 parametrization as the loss function and used a new spectral PRP conjugate gradient algorithm to optimize the inversion, and proposed a robust AVO inversion algorithm based on generalized nonconvex dictionary learning^24^.

The conventional PRPCG method is considered to have good stability and numerical performance, but it may fall into infinite loops near local minima during iteration, making the objective function unable to converge. Therefore, to ensure the inversion stability and improve the inversion results, this paper first proposed an improved PRPCG-LSRTM (IPRPCG-LSRTM) imaging method, which constructed an optimization factor to improve the iteration direction to make it fall sufficiently and can improve the iteration speed. Subsequently, a bidirectional illumination preconditioning (IP) operator was used for IPRPCG-LSRTM, and the IPRPCG-IP-LSRTM was proposed, which can solve the low-resolution problem caused by insufficient iterative gradient information.

## Methods

### Principles of LSRTM

**Figure 1:**
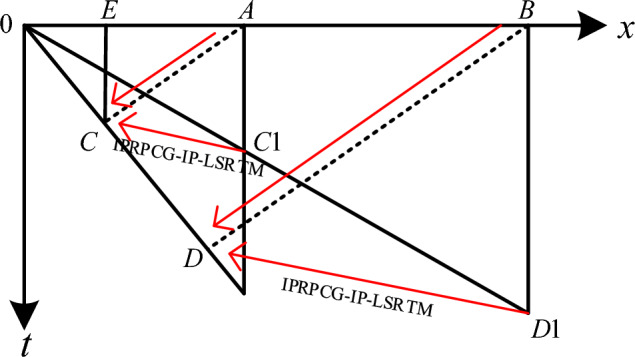
Diagram of migration principle.

LSRTM is based on traditional reverse time migration imaging and combines inversion ideas to perform migration imaging on seismic profiles. It is the migration result under the framework of least squares inversion, which corrects the migration imaging process and results. The migration principle is shown in Fig. 1:

Figure 1 is the migration principle diagram under the constant velocity model. The profile that has not been migration is $0C1D1$, and the migration profile is $0CD$. On the unmigrated profile $0C1D1$, the reflected wave generate from point $C$ directly below point $E$ is observed at point $A$ and plotted at point $C1$. The true reflection point is in the upward tilt direction of its apparent position. After using the IPRPCG-IP-LSRTM method proposed in this article, the reflection layer segment $0C1D1$ can be regressed to its real position $CD$.

Born approximation shows that:

1 $$ D(r,t|s,0) = \int {m(x)W(t) * G(r,t|x,0) * G(r,t|s,0)dx} $$

where $s$ is the source point, $x$ is the underground reflection point, and $r$ is the detection point, $D(r,t|s,0)$ is the recorded seismic data model, $m(x)$ is the reflectivity model, $W(t)$ is the source, $G(r,t|x,0)$ is Green function from source to underground reflection point, $G(r,t|s,0)$ is Green function from underground reflection point to detection point. The matrix of Born approximation is expressed as:

2 $$ d = Lm $$

where $L$ is the Born forward operator, also known as the reverse migration operator.

Since $L^{ - 1}$ is difficult to achieve, the conventional reverse time offset is simplified using $L^{T}$ instead of $L^{ - 1}$. The expression is as follows:

3 $$ m = L^{T} d $$

where $L^{T}$ denotes the adjoint operator, also known as the migration operator.

This substitution simplifies the process of solving the reverse time migration, but it has an impact on the resolution and amplitude preservation of the migration imaging. To mitigate the negative effects of this effect, the idea of least square inversion is introduced to construct the objective function for fitting and minimizing the objective function:

4 $$ E = \frac{1}{2}\left\| {Lm - d_{obs} } \right\|_{2}^{2} $$

where $d_{obs}$ is the observed seismic data.The minimization of Eq. (4) is to minimize the derivative of the objective function with respect to the model $m$:

5 $$ \frac{\partial E}{{\partial m}} = 0 $$

The equation can be solved as follows:

6 $$ m = (L^{T} L)^{ - 1} L^{T} d $$

where $L^{T} L$ is the Hessian matrix, forming the Hessian matrix is challenging, and computing the inverse of this matrix is also very difficult, especially for large 3D imaging applications. Therefore, an iterative algorithm is used to solve the above objective function.

In this paper, data reconstruction of simulated seismic data can be achieved by these equations as follows:

7 $$ \frac{1}{{v_{0} (x)^{{2}} }}\frac{{\partial^{2} u_{0} (x_{s} ,x,t)}}{{\partial t^{2} }} - \nabla^{2} u_{0} (x_{s} ,x,t) = W(t)\delta (x - x_{s} ) $$

8 $$ \frac{1}{{v_{0} (x)^{2} }}\frac{{\partial^{2} \delta u(x_{s} ,x,t)}}{{\partial t^{2} }} - \nabla^{2} \delta u(x_{s} ,x,t) = - \frac{m}{{v_{0} (x)^{2} }}\frac{{\partial^{2} }}{{\partial t^{2} }}u_{0} (x_{s} ,x,t) $$

9 $$ d(x_{s} ,x_{r} ,t) = \delta u(x_{s} ,x_{r} ,t) $$

Equation (7) is the wave field forward modeling, where $v_{0} (x)$ is the background velocity, $u_{0} \left( {x_{s} ,x,t} \right)$ is the background wave field, $\delta \left( {x - x_{s} } \right)$ is the Dirac function of the source. Equation (8) uses the background wave field and model parameter $m$ as the secondary source, the disturbed wave field $\delta u(x_{s} ,x_{r} ,t)$ is obtained. It can be seen that the solution of the disturbance wave field requires two finite difference simulations. The numerical simulation process of the Finite difference method is shown in Appendix 1. Equation (9) records the disturbance wave field at the detection point $x_{r}$ to obtain simulated data $d(x_{s} ,x_{r} ,t)$.

### Principles of IPRPCG-IP-LSRTM

In solving the objective function Eq. (4), to avoid directly solving the inverse of the Hessian matrix, the gradient method is often used for multiple indirect iterations. The conjugate gradient method has the characteristics of a small amount of calculation, high accuracy, good stability, and low storage requirements. Due to the different conjugate gradient parameters $\beta$, it can be divided into FR, PRP, HS, CD, etc. Among them, PRPCG is recognized as one of the best numerical performance methods^25^. When the algorithm generates a small step, the gradient of this objective function is close to the gradient of the previous objective function, and the search direction will automatically approach the negative gradient direction, which can effectively avoid the generation of successive small steps. Therefore, this article uses the PRPCG to find the least squares optimal solution, and the calculation formula for the parameter $\beta$ is:

10 $$ \beta_{k}^{PRP} = \frac{{g_{k}^{T} (g_{k} - g_{k - 1} )}}{{g_{k - 1}^{T} g_{k - 1} }} $$

where $g_{k} = \nabla f\left( {x_{k} } \right)$ represents the gradient operator of the objective function $f\left( x \right)$. However, the convergence of the PRPCG is relatively weak, and it may loop infinitely far from the global optimal solution, and produce a search direction that makes the objective function rise resulting in diverging scenario and stopping further computations^26^. Therefore, a parameter $\beta$ containing an optimization factor $\sigma$ is constructed in this paper to solve this problem. This factor can modify the iteration direction of $\beta$ to reduce the residual values and improve the iteration speed, as shown in Eq. (11):

11 $$ \beta_{k} { = }\frac{{g_{k}^{T} (g_{k} - g_{k - 1} )}}{{\sigma_{k} g_{k - 1}^{T} g_{k - 1} }}\,\, $$

To decrease the value of the $\beta_{k}$, the optimization factor $\sigma_{k}$ must satisfy $\sigma_{k} > 1$. so the construction of $\sigma_{k}$ is shown as follows:

12 $$ \sigma_{k} = 1 + \mu_{k} $$

In Eq. (12), $\mu_{k}$ is defined as the limiting factor of $\sigma_{k}$. The limiting factor $\mu_{k}$ is related to the current gradient and the last gradient, and we construct a function $\mu_{k}$, which is given as $\mu_{k} = f(g_{k - 1} ,g_{k} )$. According to the theory of global convergence and the direction of the fastest descending gradient, the expression of $\mu_{k}$ is constructed as Eq. (13):

13 $$ \mu_{k} = \tau \frac{{\left| {g_{k}^{T} g_{k - 1} } \right|}}{{g_{k}^{T} g_{k} }},\begin{array}{*{20}c} {} \\ \end{array} (\tau \ge 1) $$

where $\tau$ is a constant greater than or equal to one. The limiting factor $\mu_{k}$ is the fine-tuning quantity of $\beta_{k}$, which modifies the optimization factor $\sigma_{k}$ through the last gradient value and the current gradient value based on ensuring the decline of $\beta_{k}$. Then, the optimized conjugate gradient parameter $\beta_{k}$ is given as follows:

14 $$ \beta_{k} = \frac{{g_{k}^{T} (g_{k} - g_{k - 1} )}}{{(1 + \tau \frac{{\left| {g_{k}^{T} g_{k - 1} } \right|}}{{g_{k}^{T} g_{k} }})g_{k - 1}^{T} g_{k - 1} }} $$

For any $k \ge 1$, due to $1 + \tau \frac{{\left| {g_{k}^{T} g_{k - 1} } \right|}}{{g_{k}^{T} g_{k} }} > 1$, the following equation holds:

15 $$ \left| {\beta_{k} } \right| = \frac{{\left| {g_{k}^{T} (g_{k} - g_{k - 1} )} \right|}}{{(1 + \tau \frac{{\left| {g_{k}^{T} g_{k - 1} } \right|}}{{g_{k}^{T} g_{k} }})g_{k - 1}^{T} g_{k - 1} }} < \frac{{\left| {g_{k}^{T} (g_{k} - g_{k - 1} )} \right|}}{{g_{k - 1}^{T} g_{k - 1} }} = \left| {\beta_{k}^{PRP} } \right| $$

It can be seen that the optimization factor $\sigma_{k}$ can automatically adjust parameter $\beta_{k}$ to meet $\left| {\beta_{k} } \right| < \left| {\beta_{k}^{PRP} } \right|$, that is, the constructed $\sigma_{k}$ can adjust the descent direction of the gradient to automatically generate a sufficient descent direction, search along the feasible descent direction, find the feasible point to lower the objective function, determine the appropriate moving step and accelerate the convergence speed of the objective function.

In LSRTM, the gradient is the reverse time migration imaging result of the residual between simulated seismic data and observed seismic data, which also requires two finite difference calculations to calculate the background and residual wave fields:

16 $$ \frac{1}{{v_{0} (x)^{2} }}\frac{{\partial^{2} u_{0} (x_{s} ,x,t)}}{{\partial t^{2} }} - \nabla^{2} u_{0} (x_{s} ,x,t) = w\left( t \right) $$

17 $$ \frac{1}{{v_{0} (x)^{2} }}\frac{{\partial^{2} q(x_{s} ,x,t)}}{{\partial t^{2} }} - \nabla^{2} q(x_{s} ,x,t) = Lm - d_{obs} (x_{s} ,x_{r} ,t) $$

where $q(x_{s} ,x,t)$ is the residual wave field, the background wave field is a forward continuation in the time direction, and the residual wave field is a backward continuation in the time direction. Therefore, the gradient can be obtained using the following formula:

18 $$ grad = \sum\limits_{t} {\frac{1}{{v_{0} (x)^{2} }}u_{0} (x_{s} ,x,t)} \cdot q(x_{s} ,x,t) $$

The calculation of gradient is the reverse time migration of data residuals, which can be expressed in the following matrix form:

19 $$ g = L^{T} (Lm - d_{obs} ) $$

The search direction generated by the IPRPCG satisfies the sufficient descent condition and the global convergence theorem. The convergence analysis of the algorithm is shown in Appendix 2. The specific process of using IPRPCG to find the optimal solution for least-squares reverse time migration (IPRPCG-LSRTM) is shown in Table 1.

**Table 1:** The solution process of IPRPCG-LSRTM.

The input: $m = 0$: Initial value of reflection model $\varphi > 0$: Threshold for iterative data residuals, or $k_{max}$: The maximum number of iterations, its initial value $k = 0$ The output: $m$: Image results of LSRTM
Step 1: Obtain the gradient ${\text{g}}$ of the objective function: $g_{k} = L^{T} (Lm_{k - 1} - d_{obs} )\quad \quad \quad \quad (20)$ Step 2:Take conjugate gradient optimization parameters $\beta$, conjugate gradient $d$ and the iteration step $\alpha$: $\beta_{k} = \frac{{g_{k}^{T} (g_{k} - g_{k - 1} )}}{{\sigma_{k} g_{k - 1}^{T} g_{k - 1} }}\quad \quad \quad \quad (21)$ $d_{k} = - g_{k} + \beta_{k} d_{k - 1} \quad \quad \quad \quad (22)$ $\alpha_{k} = \frac{{d_{k}^{T} g_{k} }}{{(Ld_{k} )^{T} (Ld_{k} )}}\quad \quad \quad \quad (23)$ Step 3: Update the model: $m_{k} = m_{k - 1} + \alpha_{k} d_{k} \quad \quad \quad \quad (24)$ Step 4: If $g_{k} < \varphi$ or $k = k_{\max }$ is satisfied, the algorithm is terminated, output imaging results. If no, return to Step 1

IPRPCG not only retains the excellent performance of traditional PRPCG, but also makes the search direction satisfy the condition that it is always the descent direction of the objective function, which has full descent, good global convergence performance and numerical performance. The accurate descent direction can make the data residuals smaller, so it has a great improvement in the computational accuracy and imaging resolution.

As the conventional migration operator is only the transpose of the forward operator, the illumination of some underground structures, especially deep and high-speed salt bodies, will be uneven, or even unable to image^27^. The Hessian operator can characterize the distribution of underground lighting energy, and its Inverse matrix can compensate for the uneven underground lighting phenomenon, helping us to get clear images of deep and sub-salt structures^28^. However, it is difficult to calculate the inverse of the full Hessian matrix under the current computing power. Considering that the Hessian matrix is a matrix with dominant principal diagonal elements, its diagonal elements can be used to approximate the Hessian operator, and then its Inverse matrix can be used as a precondition operator in the implementation of LSRTM^29^, which can also improve the imaging resolution. Therefore, this paper introduces the Illumination precondition operator $P = H^{ - 1}$ in the optimization parameters $\beta_{k}$ and conjugate gradient $d_{k}$, whose approximation matrix is :

25 $$ H \approx H(a) = {\text{Re}} \left\{ {\sum\limits_{w} {w^{4} \sum\limits_{{x_{s} }} {\left[ {\left| {f_{s} (w)} \right|^{2} \left| {G(x_{s} ,x,w)} \right|^{2} \cdot \sum\limits_{r} {\left| {G(x,r,w)} \right|^{2} } } \right]} } } \right\} $$

where $H(a)$ is the approximately linear part of the Hessian operator, ${\text{Re}} \left\{ {} \right\}$ means taking the real part for the complex numbers in parentheses, $w$ is the angular frequency, $x_{s}$ denotes the location of the source point, $r$ denotes the location of the detector point, $x$ denotes the location of any point in the subsurface, $f_{s} \left( w \right)$ is the frequency domain expression of the source , $G(x_{s} ,x,w)$ is the Green's function of the source, and $G(x,r,w)$ is the Green's function of the checkpoint.

Then the optimized parameters $\varepsilon_{k}$ and conjugate gradient $h_{k}$ with IP are shown follows:

26 $$ \varepsilon_{k} { = }\frac{{g_{k}^{T} P(g_{k} - g_{k - 1} )}}{{\sigma_{k} g_{k - 1}^{T} Pg_{k - 1} }} $$

27 $$ h_{k} = - Pg_{k} + \varepsilon_{k} h_{k - 1} $$

**Figure 2:**
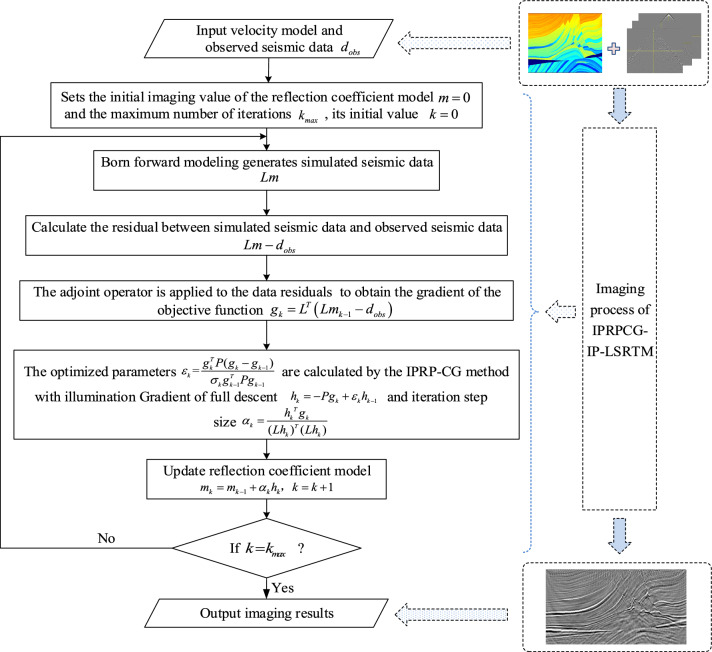
The flow chart of IPRPCG-IP-LSRTM.

Compared with the CG method, the proposed method IPRPCG-IP only adds a precondition operator and some dot product calculation at the beginning of the calculation, which can be ignored compared with LSRTM, but it can greatly improve the migration imaging effect. The overall implementation flow chart of IPRPCG-IP-LSRTM proposed in this paper is shown in Fig. 2.

## Experimental results and analysis

### Noise resistance test

**Figure 3:**
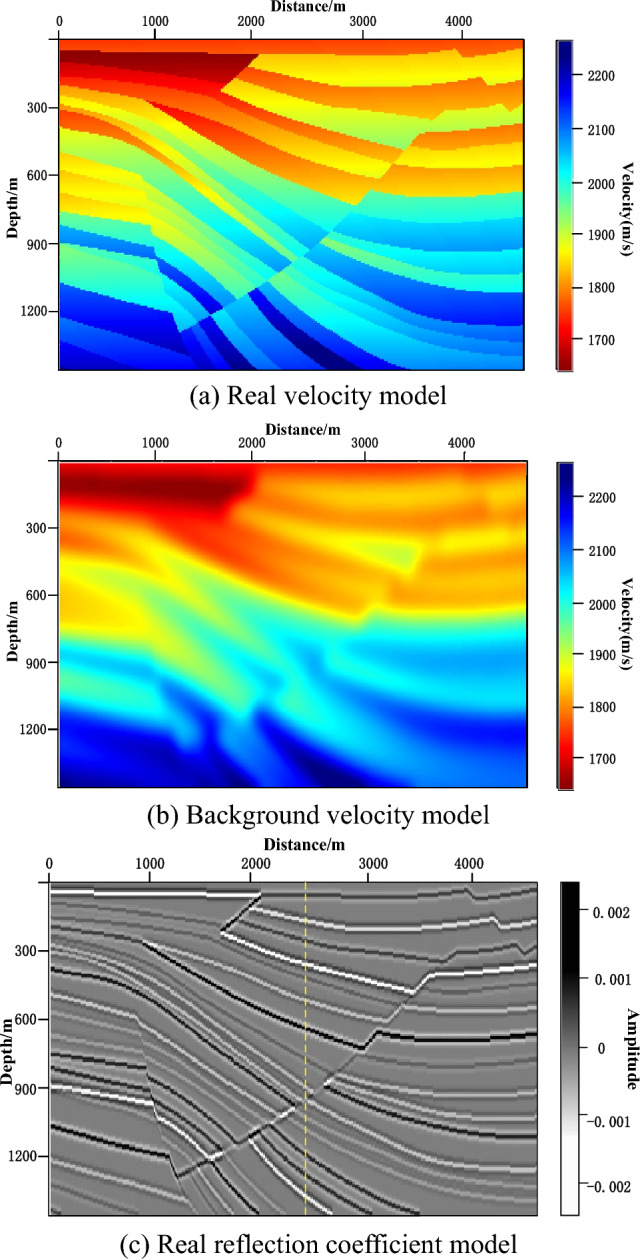
Sigsbee2B truncation velocity model.

Sigsbee2B truncation model was used for testing the noise resistance of PRPCG-LSRTM, IPRPCG-LSRTM, and IPRPCG-IP-LSRTM imaging. The velocity models were shown in Fig. 3. Figure 3a was the real velocity model, and Fig. 3b was the background velocity model, which was obtained by real velocity model smoothing. The real reflection coefficient model was shown in Fig. 3c, and the detailed test parameters were shown in Table 2.

**Table 2:** Test parameters for sigsbee2B truncation model.

Model parameter	Mesh	Rectangular grid with a grid size of 600 × 200 and a grid spacing of 7.62 *m*
	Velocity distribution	[1700 2200] *m/s*, Please refer to Fig. 3a for details
Observation system	Position	At the surface
	Source	The Ricker wavelet with a main frequency of 20* Hz* excited a total of 60 shots, with the first shot at a position of 38.1* m* and a shot interval of 76.2* m*. Each shot received a total of 600 shots
Seismic forward modeling parameters	Wave field continuation	Finite Difference
	Propagation time	Recorded duration of 2.5*s*, sampling interval of 1.0*ms*
	Wave equation	Acoustic wave Wave equation, assuming the density is constant
	Boundary	Spongy boundary condition
Inversion parameters	Initial model	The smoothed background velocity model was shown in Fig. 3b for details
	Objective function solving	IPRPCG-IP method

**Figure 4:**
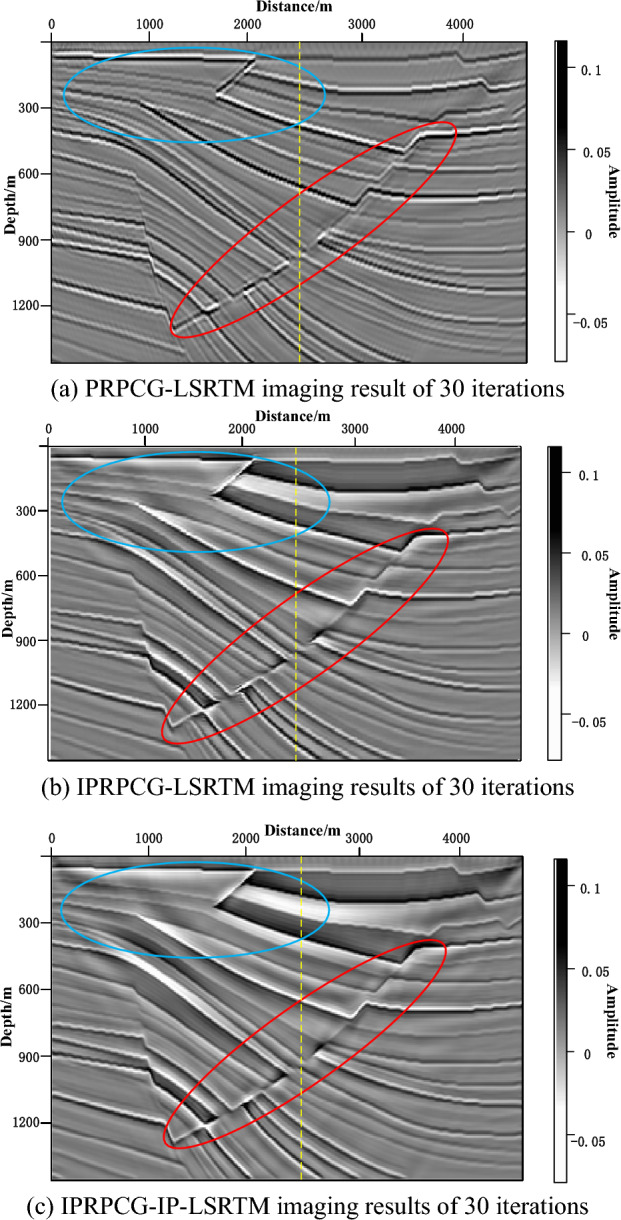
Sigsbee2B truncated model imaging results.

PRPCG-LSRTM, IPRPCG-LSRTM and IPRPCG-IP-LSRTM imaging methods were employed on Sigsbee2B truncation model, and the three methods had been used for 30 iterations. The imaging results were shown in Fig. 4, where Fig. 4a was the PRPCG-LSRTM imaging results, Fig. 4b was the IPRPCG-LSRTM imaging results, and Fig. 4c was the IPRPCG-IP-LSRTM imaging results. From the above three migration imaging results, it can be seen that the PRPCG-LSRTM imaging energy was unbalanced and more serious low-frequency noise still remained. Comparing with PRPCG-LSRTM, IPRPCG-LSRTM method imaging of Fig. 4b, the noise in the imaging profile was suppressed, the regional energy was compensated, and the tomographic part was clearer, and the effect was better than that of PRPCG-LSRTM, which proved the effectiveness of IPRPCG-LSRTM. IPRPCG-IP-LSRTM imaging of Fig. 4c compared with IPRPCG-LSRTM of Fig. 4b, the low-frequency noise was further suppressed, the profile amplitude was more balanced, the signal-to-noise ratio was higher, the wavefield energy was more balanced, and the imaging quality was improved significantly, which can be seen in the red and blue circle of Fig. 4a–c.

To evaluate the image quality of the experimental results more rigorously, Table 3 quantitatively compared the imaging results of the sigsbee2B truncated model using Peak Signal to Noise Ratio (PSNR) and Structural Similarity (SSIM). From the data in the table, it can be seen that the imaging results of IPRPCG-LSRTM iteration 30 times increased PSNR and SSIM values compared to PRPCG-LSRTM iteration 30 times, indicating a significant improvement in imaging quality. However, IPRPCG-IP-LSRTM iteration 30 times has the highest PSNR and SSIM values, indicating the highest image quality generated.

**Table 3:** Image quality evaluation of sigsbee2B truncation model imaging results.

Images generated by different methods	Evaluation indicators	
	PSNR/dB	SSIM
PRPCG-LSRTM imaging result of 30 iterations	15.0346	0.5225
IPRPCG-LSRTM imaging results of 30 iterations	16.0895	0.6738
IPRPCG-IP-LSRTM imaging results of 30 iterations	17.2597	0.7862

**Figure 5:**
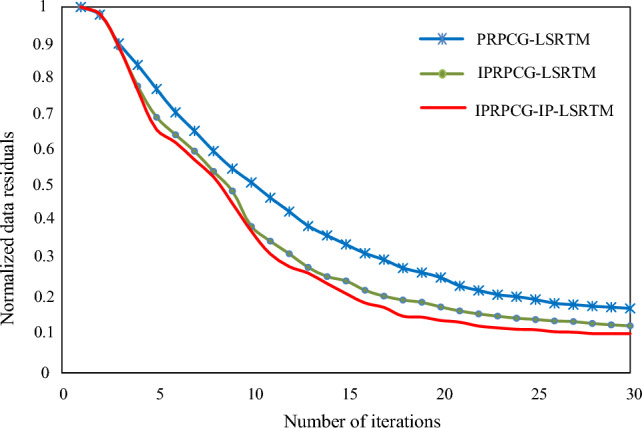
Comparison of residual convergence curves for 30 iterations of data.

Figure 5 showed the comparison of convergence curves for 30 iterations of the three methods using PRPCG-LSRTM, IPRPCG-LSRTM, and IPRPCG-IP-LSRTM.

It can be seen from the Fig. 5 that the three methods were stable from the 15th iterations. IPRPCG-IP-LSRTM (red) converged the fastest in the three methods, which improved the computational efficiency. The residual value of IPRPCG-IP-LSRTM was the smallest, so the imaging result was the closest to the real structure, the accuracy of IPRPCG-IP-LSRTM imaging was the best.

Table 4 showed the comparison of the program running time of PRPCG-LSRTM, IPRPCG-LSRTM, and IPRPCG-IP-LSRTM with 30 iterations. It can be seen that the program running time of IPRPCG-LSRTM with 30 iterations was the shortest, which improved the computational efficiency by about 24.73% compared to PRPCG-LSRTM. After adding IP, the program running time of IPRPCG-IP-LSRTM was extended compared to IPRPCG-LSRTM due to the addition of a preconditioned operator and some point multiplication calculations at the beginning of the calculation. However, compared to PRPCG-LSRTM, the program running time of IPRPCG-IP-LSRTM with 30 iterations was significantly reduced, and the computational efficiency was improved by about 23.59%.

**Table 4:** Comparison of program running times of different algorithms.

Algorithm	Program running time of 30 iterations/s
PRPCG-LSRTM	153,061.953812
IPRPCG-LSRTM	153,061.953812
IPRPCG-IP-LSRTM	116,954.638908

**Figure 6:**
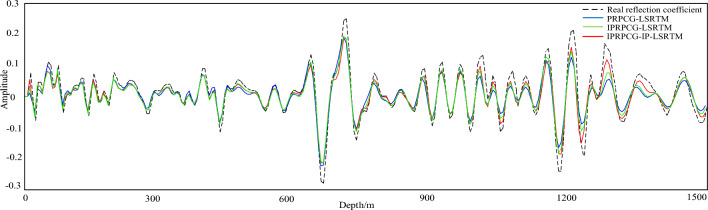
Comparison of single-channel imaging curves at a distance of 2400 m.

To compare the amplitude fidelity of PRPCG-LSRTM, IPRPCG-LSRTM, and IPRPCG-IP-LSRTM, single-channel imaging data at a distance of 2400m (indicated by the yellow dashed line) were extracted from the profiles shown in Figs. 3c, and 4a–c. for comparison, as shown in Fig. 6. From the figure, it can be seen that the effect of IPRPCG-LSRTM (green) was better than that of PRPCG-LSRTM (blue), but they only had good amplitude fidelity in the shallow layer, and there was a significant difference in the true reflection coefficient (black dashed line) in the middle and deep layers. And the amplitude of IPRPCG-IP-LSRTM (red) was closer to the real reflection coefficient, especially in the middle and deep layers, indicating that IPRPCG-IP-LSRTM could better compensate for the amplitude and illumination of energy attenuation caused by geometric diffusion or absorption attenuation in the middle and deep layers, thus it had higher amplitude preservation.

**Figure 7:**
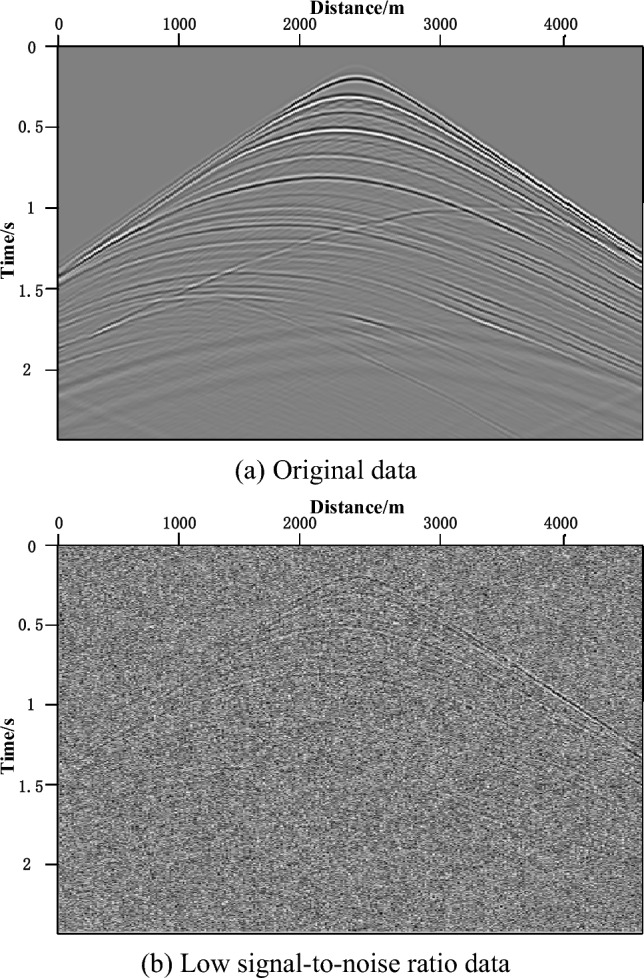
The 30th shot data without and with noise.

Artificially adding random noise to Sigsbee2B truncation model to form a data volume with low signal-to-noise ratio, which can be seen in Fig. 7.

Figure 7a was the original shot data and Fig. 7b was the one of the shots with low signal-to-noise data. PRPCG-LSRTM and IPRPCG-IP-LSRTM were employed on the low signal-to-noise data.

**Figure 8:**
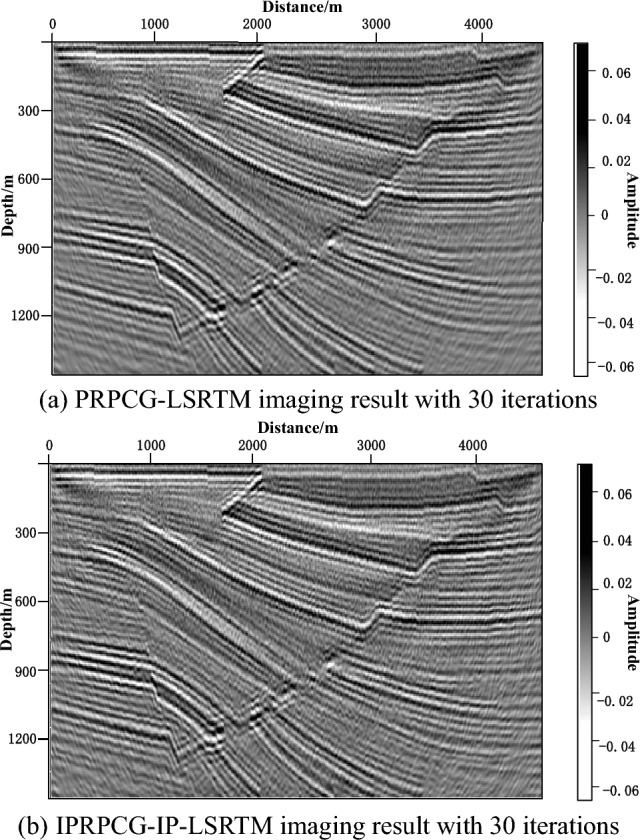
Low signal-to-noise ratio data imaging results of Sigsbee2B.

Figure 8 was the imaging results of the Low signal-to-noise ratio shot data of Sigsbee2B. Figure 8a was the imaging results of PRPCG-LSRTM with 30 iterations, and Fig. 8b was the imaging results of IPRPCG-IP-LSRTM with 30 iterations. We can be seen that both contained migration artifacts caused by the noise, but the low-frequency noise was suppressed better by IPRPCG-IP-LSRTM in Fig. 8b, and the imaging energy was more balanced, and the imaging resolution was higher.

### Accuracy and convergence test

**Figure 9:**
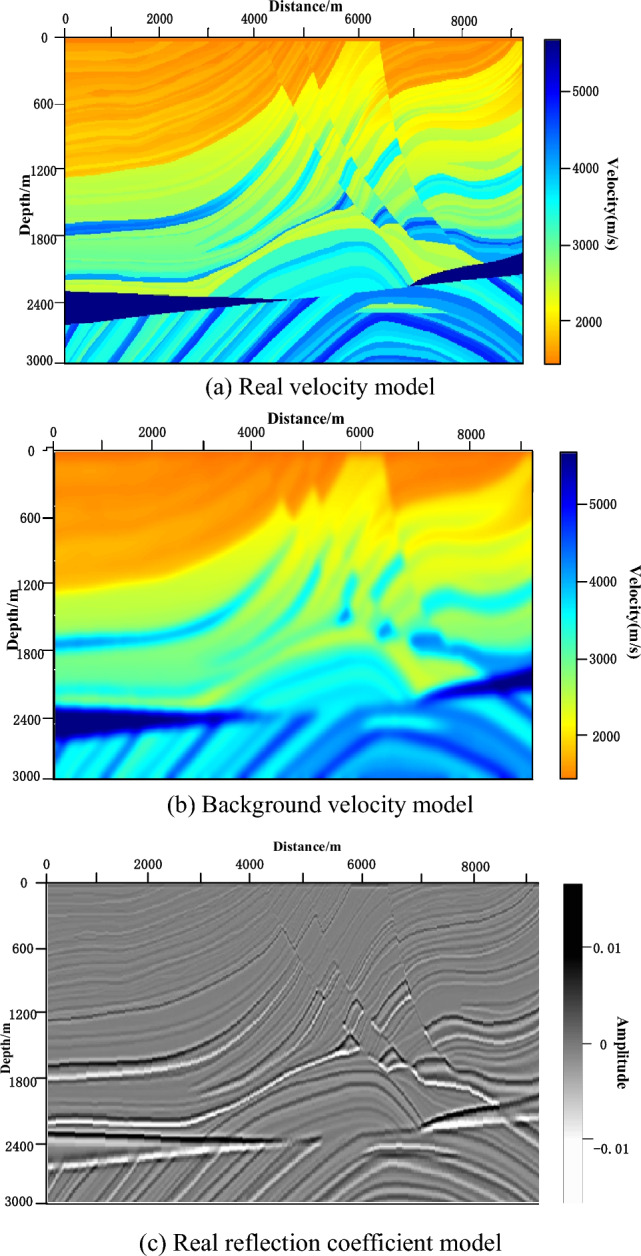
Marmousi velocity model.

Marmousi model was employed to test the convergence and accuracy of RTM, PRPCG-LSRTM, and IPRPCG-IP-LSRTM proposed in this paper, which can be shown in Fig. 9. Figure 9a was Marmousi's real velocity model. The velocity range was shown as the scale. Figure 9b was the background velocity model obtained by Marmousi's real velocity model smoothing. The detailed test parameters were shown in Table 5.

**Table 5:** Test parameters for Marmousi model.

Model parameter	Mesh	Rectangular grid with a grid size of 767 × 250 and a grid spacing of 12 *m*
	Velocity distribution	[2000 5000] *m/s*, Please refer to Fig. 9a for details
Observation system	Position	At the surface
	Source	The Ricker wavelet with a main frequency of 20 *Hz* excited a total of 64 shots, with the first shot at a position of 60 *m* and a shot interval of 144 *m*. Each shot received a total of 767 shots
Seismic forward modeling parameters	Wave field continuation	Finite Difference
	Propagation time	Recorded duration of 4 *s*, sampling interval of 1.0 *ms*
	Wave equation	Acoustic wave Wave equation, assuming the density is constant
	Boundary	Spongy boundary condition
Inversion parameters	Initial model	The smoothed background velocity model was shown in Fig. 9b for details
	Objective function solving	IPRPCG-IP method

**Figure 10:**
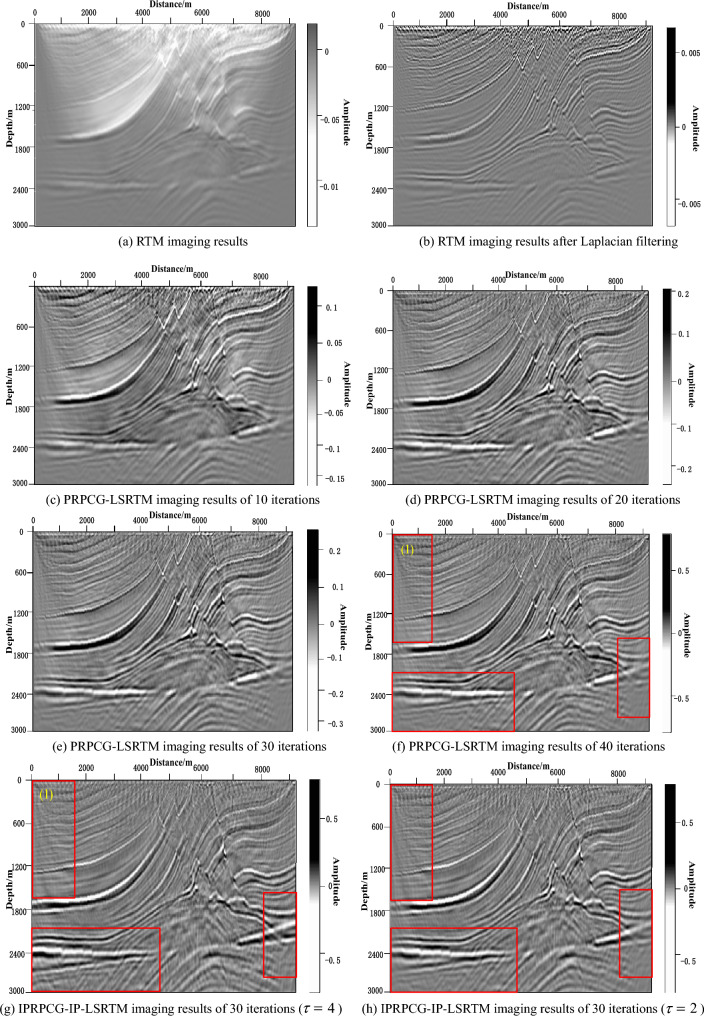
Marmousi model migration imaging results of different methods.

The migration imaging results of the above methods were shown in Fig. 10. Figure 10a showed the RTM imaging results of Marmousi. It can be seen that there was the serious low-frequency noise in the imaging results, the shallow imaging was week and the deep area imaging was not clear. Figure 10b showed the imaging result after Laplacian filtering based on RTM, and it can be seen from the figure that there was the serious noise in shallow surface area.

Figure 10c–f showed the migration imaging results of 10, 20, 30 and 40 iterations based on PRPCG-LSRTM. It can be seen that with the increase in the number of iterations, the imaging result become better and better, the source effect decreased, the energy become more balanced, the imaging on both sides and deep layers become clearer, the event energy was restored, the noise was suppressed, and the imaging resolution was higher.

Figure 10g showed the imaging results of IPRPCG-IP-LSRTM with 30 iterations. Compared with PRPCG-LSRTM imaging results with 40 iterations of Fig. 10f, the imaging energy is more uniform and the imaging of details was clearer. And it can be seen from Fig. 10f, g that the IPRPCG-IP-LSRTM migration imaging result with 30 iterations was better than PRPCG-LSRTM with 40 iterations, especially the areas in the red boxes.

Figure 10g showed the imaging results of IPRPCG-IP-LSRTM with 30 iterations when the parameter $\tau = 4$, and Fig. 10h showed the imaging results of IPRPCG-IP-LSRTM with 30 iterations when the parameter $\tau = 2$. After experiments with parameter $\tau$ in the optimization factor $\sigma$, it was found that the best results are obtained when parameter $\tau = 4$ of Marmousi model. Comparing Fig. 10g, h, it can be seen that the imaging of depth boundary with less data and deeper layers of Fig. 10g was clearer, with more balanced energy and higher resolution.

**Figure 11:**
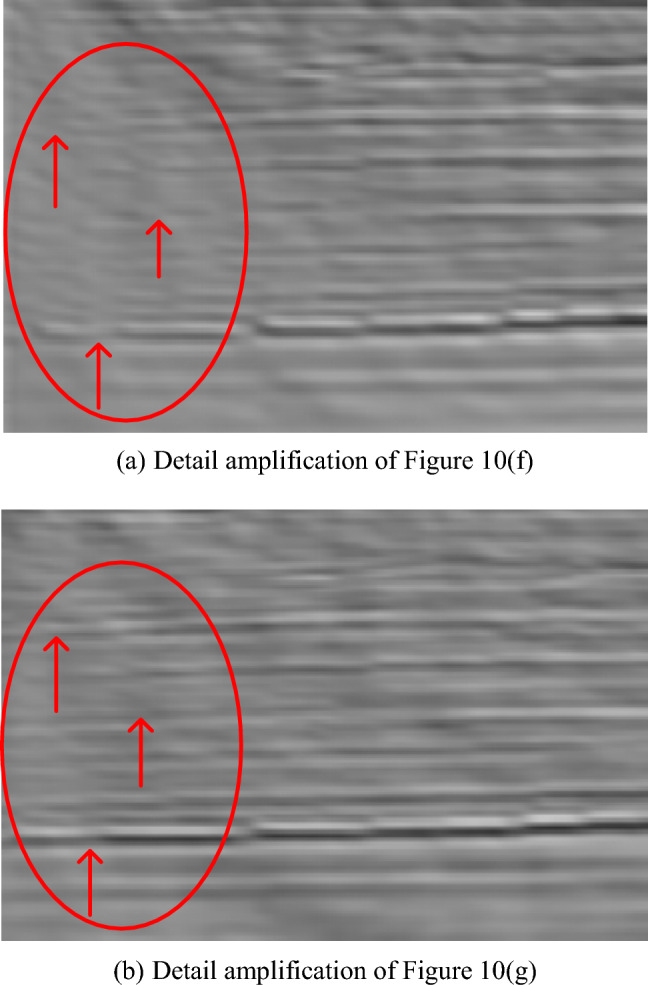
Comparison of image details of IPRPCG-LSRTM and PRPCG-LSRTM.

We compared the magnified details in the red box (1) of Fig. 10f, g, they were shown in Fig. 11. Figure 11a showed the enlarged detail image of the imaging results in the red box (1) of Figs. 10f, and 11b showed the enlarged image of the imaging results in the red box (1) of Fig. 10g. Magnifying the imaging details in the red circle, it can be seen that the geological structure (horizon) of PRPCG-LSRTM had a fracture and no imaging, while the layer structure (event) was continuous and clear in Fig. 11b. It can be seen that the IPRPCG-IP-LSRTM method proposed in this paper can improve the resolution of the migration imaging result effectively.

Table 6 showed the image quality evaluation of the Marmousi model's imaging results using PSNR and SSIM indicators. It can be seen from the table that the image quality of RTM had improved after Laplace filtering, and as the number of iterations increases from 10 to 40, the imaging effect of PRPCG-LSRTM also improved. The imaging quality of the IPRPCG-IP-LSRTM proposed in this article was already better at 30 iterations than that of PRPCG-LSRTM at 40 iterations, and the imaging effect of IPRPCG-IP-LSRTM was the best when $\tau = 4$, indicating the effectiveness of the algorithm proposed in this article.

**Table 6:** Image quality evaluation of Marmousi model imaging results.

Images generated by different methods	Evaluation indicators	
	PSNR/dB	SSIM
RTM	13.7780	0.4187
RTM after Laplace filtering	13.9743	0.4963
PRPCG-LSRTM with 10 iterations	14.4319	0.5218
PRPCG-LSRTM with 20 iterations	14.8507	0.5607
PRPCG-LSRTM with 30 iterations	15.6323	0.6142
PRPCG-LSRTM with 40 iterations	16.4251	0.6733
IPRPCG-IP-LSRTM with 30 iterations ($\tau = 2$)	17.3265	0.7331
IPRPCG-IP-LSRTM with 30 iterations ($\tau = 4$)	17.5318	0.7502

**Figure 12:**
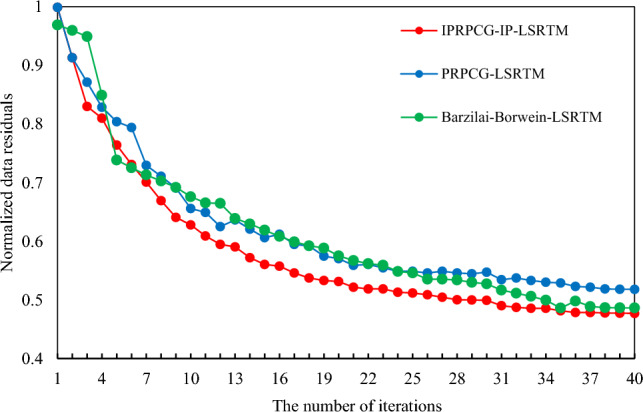
Comparison of residual convergence curves for normalized data.

Figure 12 showed a comparison of the convergence curves of normalized data residuals using the Barzilai Borwein algorithm for least squares reverse time migration (Barzilai-Borwein-LSRTM) based on the Marmousi model, compared to PRPCG-LSRTM and IPRPCG-IP-LSRTM with 40 iterations. From the figure, it can be seen that the normalized data residuals of Barzilai-Borwein-LSRTM decreased rapidly during 1–5 iterations, and the effect of Barzilai-Borwein-LSRTM was similar to that of PRPCG-LSRTM during 10–25 iterations. At 26 to 40 iterations, the normalized data residuals of Barzilai-Borwein-LSRTM were smaller than those of PRPCG-LSRTM. However, from beginning to end, the normalized data residuals of Barzilai-Borwein-LSRTM and PRPCG-LSRTM were not lower than those of IPRPCG-IP-LSRTM. This meant that the convergence accuracy of the IPRPCG-IP-LSRTM proposed in this paper was the highest, and the imaging data was closer to real data.

Table 7 showed the comparison of the computational efficiency of IPRPCG-IP-LSRTM and PRPCG-LSRTM. From the data in the table, it can be seen that the normalized residual values of IPRPCG-IP-LSRTM decreased faster than PRPCG-LSRTM, with an average of about 6.55%. Additionally, the program running time of IPRPCG-IP-LSRTM was shorter than PRPCG-LSRTM, with an average improvement in computational efficiency of about 22.45%. This demonstrated the superiority and effectiveness of IPRPCG-IP-LSRTM in terms of imaging accuracy and descent speed.

**Table 7:** Comparison of computational efficiency.

Item	Iteration number				
	5	10	20	30	40
Normalized data residual value of PRPCG-LSRTM	0.804052	0.655863	0.570409	0.54737	0.51804
Normalized data residual value of IPRPCG-IP-LSRTM	0.764052	0.627863	0.531409	0.49937	0.47704
Reduced residual value	0.0400	0.0280	0.0389	0.0480	0.0410
Improvement percentage (%)	4.97	4.27	6.82	8.77	7.91
Average improvement percentage (%)			6.55		
Program running time of PRPCG-LSRTM/*s*	34,589.763707	68,221.798763	134,833.735890	310,722.314877	448,659.640109
Program running time of IPRPCG-IP-LSRTM/*s*	27,529.992934	53,697.377806	102,986.007473	235,900.381454	347,531.757228
Improved computing efficiency (%)	20.41	21.29	23.62	24.38	22.54
Average improvement percentage (%)			22.45		

**Figure 13:**
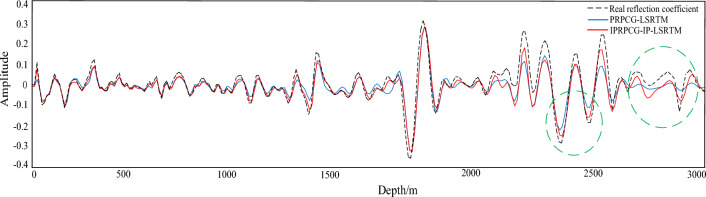
Comparison of single-channel imaging curves at a distance of 1200 m.

For observing the imaging details, single-trace data of migration imaging was extracted. Figure 13 showed the single-channel amplitude comparison between the imaging results of the 30th iteration of PRPCG-LSRTM, IPRPCG-IP-LSRTM, and the real reflection coefficient at a distance of 1200 m. From the figure, it can be seen that the imaging effect of IPRPCG-IP-LSRTM was better than PRPCG-LSRTM, especially when there was a high-speed layer at a depth of 2400–2600 m, the amplitude curve of IPRPCG-IP-LSRTM could better match the true reflection coefficient. The amplitude energy of IPRPCG-IP-LSRTM was stronger than that of PRPCG-LSRTM. At the depth of 2700–3000 m, the amplitude energy of PRPCG-LSRTM was greatly reduced, while the amplitude energy of IPRPCG-IP-LSRTM was still very strong, closer to the amplitude energy of the real reflection coefficient, as shown in the green dashed circle in Fig. 13.

## Discussions

PRPCG had not been widely used because of its good numerical performance and relatively weak convergence, which may produce a search direction that makes the objective function rise and thus fail to converge. With the work done, we proposed a method that improved the PRPCG and incorporated the IP operator for the combination. Compared with earlier work, Zhao et al.^30^ used the original PRP conjugate gradient method to solve the sparse joint inverse objective generalization, and we solved the problem that the PRP method may not converge globally. Regarding the earlier finding of Jin et al.^31^ that effective tectonic information could not be obtained due to uneven illumination when performing offset imaging in complex tectonic zones in the subsurface, we used IP operators to compensate for illumination and improve image resolution. In addition, recent work by Rong et al.^32^ showed that it was possible to use deep learning to obtain low-illumination regions of geological models and recovered complex geological formations in the region.

## Conclusions

The advantages of IPRPCG-IP-LSRTM proposed in this paper were that it can effectively improve the migration imaging resolution and computational efficiency. The method started from the gradient descent direction, constructed the optimization factor, improved the conjugate gradient parameters, added the illumination preconditioning operator, accelerated the convergence speed, reduced the normalized data residual values, and effectively improved the computational efficiency and imaging accuracy.

According to the experiments in this paper, the method IPRPCG-IP-LSRTM proposed can reduce the average residuals of normalized data by about 6.55%, which verified the effectiveness and feasibility of the algorithm. The disadvantage was that due to the huge computational power of LSRTM, a suitable number of iterations should be selected when using the IPRPCG-IP-LSRTM method in practical applications. Finally, combining our method with deep learning and intelligent optimization was our further research.

## Figures and Tables

**Figure 14:**
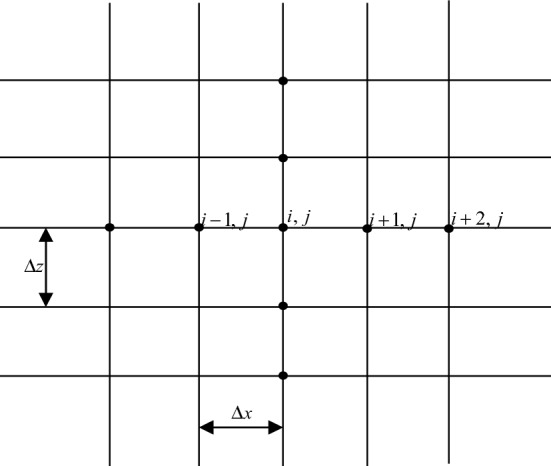
Grid-based spatial model.

## Appendix 1: Numerical simulation of finite difference method

The two-dimensional constant density acoustic Wave equation can describe the propagation of acoustic waves in Seismic waves in underground media:

28 $$ \frac{{\partial^{2} u}}{{\partial x^{2} }} + \frac{{\partial^{2} u}}{{\partial z^{2} }} = \frac{1}{{v^{2} }}\frac{{\partial^{2} u}}{{\partial t^{2} }} $$

where $u$ is the wave field function $u\left( {x,z,t} \right)$, $v$ is the medium velocity $v(x,z)$, $x$ and $z$ are the spatial coordinate components, $x$ represents the horizontal direction, $z$ represents the vertical direction, and ${\text{t}}$ is the temporal coordinate component.

The finite difference method is used to Discretization the numerical solution of Eq. (28). The wave field space should first be Discretization. Therefore, the spatial model needs to be meshed, as shown in Fig. 14:

If the grid spacing after Discretization in $x$ and $z$ directions is $\Delta x$ and $\Delta z$, and $\Delta t$ is the time sampling step, $x = i\Delta x$, $z = j\Delta z$, $t = n\Delta t$ can be obtained. Where $i$, $j$, and $n$ are integers. Assuming $u_{i,j}^{k}$ represents the wave field value at time $k$ at point $(i,j)$, expand $(x,z)$ at time $k$ using Taylor's formula:

29 $$ u_{i,j}^{k + 1} = u_{i,j}^{k} + \Delta t\frac{\partial u}{{\partial t}}\left| {_{t = k*\Delta t} } \right. + \frac{1}{2}\Delta t^{2} \frac{{\partial^{2} u}}{{\partial t^{2} }}\left| {_{t = k*\Delta t} } \right. + o(\Delta t^{2} ) $$

Expand $u_{i,j}^{k - 1}$ at time $k$ at point $(i,j)$ using Taylor's expansion:

30 $$ u_{i,j}^{k - 1} = u_{i,j}^{k} - \Delta t\frac{\partial u}{{\partial t}}\left| {_{t = k*\Delta t} } \right. + \frac{1}{2}\Delta t^{2} \frac{{\partial^{2} u}}{{\partial t^{2} }}\left| {_{t = k*\Delta t} } \right. + o(\Delta t^{2} ) $$

Add Eqs. (29) and (30) above, omit high-order small quantities, and organize the second-order time partial differential of point $(i,j)$ and time $k$ as follows:

31 $$ \frac{{\partial^{2} u}}{{\partial t^{2} }} = \frac{{u_{i,j}^{k + 1} - 2u_{i,j}^{k} + u_{i,j}^{k - 1} }}{{\Delta t^{2} }} $$

Similarly, the second-order space partial differential at point $(i,j)$ and time $k$ can be obtained as follows:

32 $$ \frac{{\partial^{2} u}}{{\partial x^{2} }} = \frac{{u_{i + 1,j}^{k} - 2u_{i,j}^{k} + u_{i - 1,j}^{k} }}{{\Delta x^{2} }} $$

33 $$ \frac{{\partial^{2} u}}{{\partial z^{2} }} = \frac{{u_{i,j + 1}^{k} - 2u_{i,j}^{k} + u_{i,j - 1}^{k} }}{{\Delta z^{2} }} $$

The above equation replaces the derivative of the second-order partial differential with the difference of the wave field values of discrete grid points. By substituting Eqs. (31), (32), and (33) into Eqs. (28), (34) can be obtained:

34 $$ u_{i,j}^{k + 1} = \frac{{v^{2} \Delta t^{2} }}{{\Delta x^{2} }}(u_{i + 1,j}^{k} + u_{i - 1,j}^{k} + 2u_{i,j}^{k} ) + \frac{{v^{2} \Delta t^{2} }}{{\Delta z^{2} }}(u_{i,j + 1}^{k} + u_{i,j - 1}^{k} + 2u_{i,j}^{k} ) + 2u_{i,j}^{k} - u_{i,j}^{k - 1} $$

Similarly, the difference scheme of the second-order Time derivative and the space $2N$ order acoustic equation is as follows:

35 $$ u_{i,j}^{k + 1} = 2u_{i,j}^{k} - u_{i,j}^{k - 1} + \frac{{v^{2} \Delta t^{2} }}{{\Delta x^{2} }}\left[ {\sum\limits_{m = 1}^{N} {a_{m} (u_{i + m,j}^{k} } + u_{i - m,j}^{k} ) + a_{0} u_{i,j}^{k} } \right] + \frac{{v^{2} \Delta t^{2} }}{{\Delta z^{2} }}\left[ {\sum\limits_{m = 1}^{N} {a_{m} } (u_{i,j + m}^{k} + u_{i,j - m}^{k} ) + a_{0} u_{i,j}^{k} } \right] $$

Among them, $a_{m}$ is the $2N$ order accuracy center difference coefficient, determined by Eq. (36):

36 $$ \begin{aligned} & \left( {\begin{array}{*{20}c} 1 & {2^{2} } & \cdots & {N^{2} } \\ 1 & {2^{4} } & \cdots & {N^{4} } \\ {} & {} & \cdots & {} \\ 1 & {2^{2N} } & \cdots & {N^{2N} } \\ \end{array} } \right)\left( {\begin{array}{*{20}c} {a_{1} } \\ {a_{2} } \\ \cdots \\ {a_{N} } \\ \end{array} } \right) = \left( {\begin{array}{*{20}c} 1 \\ 0 \\ \cdots \\ 0 \\ \end{array} } \right) \\ {\kern 1pt} & \quad a_{0} = - 2\sum\limits_{l}^{N} {a_{l} } \\ \end{aligned} $$

By solving Eq. (36), it can be concluded that:

When $$N = 1$$, $$a_{1} = 1{\kern 1pt} {\kern 1pt} {\kern 1pt}$$, $${\kern 1pt} a_{0} = - 2{\kern 1pt}$$;

When $$N = 2$$, $$a_{1} = \frac{4}{3}$$,$$a_{2} = - \frac{1}{12}{\kern 1pt} {\kern 1pt}$$,$$a_{0} = - \frac{5}{2}{\kern 1pt}$$;

When $$N \ge 3$$,$$a_{l} = \frac{( - 1)}{{l^{2} }} \cdot \frac{{\prod\limits_{i = 1,i \ne l}^{N} {i^{2} } }}{{\prod\limits_{i = 1}^{l - 1} {(l^{2} - i^{2} )\prod\limits_{i = l + 1}^{N} {(i^{2} - l^{2} )} } }},{\kern 1pt} {\kern 1pt} {\kern 1pt} {\kern 1pt} {\kern 1pt} {\kern 1pt} l = 1,2, \cdots ,N$$;

When $$N \to \infty$$, $$a_{l} = ( - 1)^{l + 1} \cdot \frac{2}{{l^{2} }}$$。

## Appendix 2: Convergence analysis of IPRPCG

Making the inner product of both ends of Eq. (22) with $g_{k}$ and using Eq. (11) yields:

37 $$ \frac{{g_{k}^{T} d_{k} }}{{\left\| {g_{k} } \right\|^{2} }} = - 1 + \frac{{g_{k}^{T} \left( {g_{k} - g_{k - 1} } \right)}}{{\sigma_{k} \left\| {g_{k - 1} } \right\|^{2} }}\frac{{g_{k}^{T} d_{k - 1} }}{{\left\| {g_{k} } \right\|^{2} }} = - 1 + \frac{{\left\| {g_{k} } \right\|^{2} \left( {1 - \frac{{\left\| {g_{k - 1} } \right\|}}{{\left\| {g_{k} } \right\|}}\cos \theta_{k} } \right)}}{{(1 + \tau \frac{{\left\| {g_{k - 1} } \right\|}}{{\left\| {g_{k} } \right\|}}\cos \theta_{k} )\left\| {g_{k - 1} } \right\|^{2} }}\frac{{g_{k}^{T} d_{k - 1} }}{{\left\| {g_{k} } \right\|^{2} }} = - 1 + \frac{{1 - \frac{{\left\| {g_{k - 1} } \right\|}}{{\left\| {g_{k} } \right\|}}\cos \theta_{k} }}{{1 + \tau \frac{{\left\| {g_{k - 1} } \right\|}}{{\left\| {g_{k} } \right\|}}\cos \theta_{k} }}\frac{{g_{k}^{T} d_{k - 1} }}{{\left\| {g_{k - 1} } \right\|^{2} }} $$

For any $\tau \ge 1$ and $k \ge 1$, let $\theta_{k}$ be the angle between $g_{k}$ and $g_{k - 1}$. From Eq. (14), we have:

38 $$ \left| {\beta_{k} } \right| = \frac{{\left| {g_{k}^{T} \left( {g_{k} - g_{k - 1} } \right)} \right|}}{{(1 + \tau \frac{{\left| {g_{k}^{T} g_{k - 1} } \right|}}{{g_{k}^{T} g_{k} }})g_{k - 1}^{T} g_{k - 1} }} = \frac{{\left| {\left\| {g_{k} } \right\|^{2} \left( {1 - \frac{{\left\| {g_{k - 1} } \right\|}}{{\left\| {g_{k} } \right\|}}\cos \theta_{k} } \right)} \right|}}{{\left( {1 + \tau \frac{{\left| {g_{k}^{T} g_{k - 1} } \right|}}{{g_{k}^{T} g_{k} }}\cos \theta_{k} } \right)g_{k - 1}^{T} g_{k - 1} }} \le \frac{{\left| {\left\| {g_{k} } \right\|^{2} \left( {1 + \frac{{\left\| {g_{k - 1} } \right\|}}{{\left\| {g_{k} } \right\|}}\left| {\cos \theta_{k} } \right|} \right)} \right|}}{{\left( {1 + \tau \frac{{\left| {g_{k}^{T} g_{k - 1} } \right|}}{{g_{k}^{T} g_{k} }}\cos \theta_{k} } \right)g_{k - 1}^{T} g_{k - 1} }} \le \frac{{\left\| {g_{k} } \right\|^{2} }}{{\left\| {g_{k - 1} } \right\|^{2} }} $$

From Eq. (38), we know that $\frac{{1 - \frac{{\left\| {g_{k - 1} } \right\|}}{{\left\| {g_{k} } \right\|}}\cos \theta_{k} }}{{1 + \tau \frac{{\left\| {g_{k - 1} } \right\|}}{{\left\| {g_{k} } \right\|}}\left| {\cos \theta_{k} } \right|}} \le 1$, so we can get:

39 $$ - 1 - \frac{{\left| {g_{k}^{T} d_{k - 1} } \right|}}{{\left\| {g_{k - 1} } \right\|^{2} }} \le \frac{{g_{k}^{T} d_{k} }}{{\left\| {g_{k} } \right\|^{2} }} \le - 1 + \frac{{\left| {g_{k}^{T} d_{k - 1} } \right|}}{{\left\| {g_{k - 1} } \right\|^{2} }} $$

Also from $\left| {g_{k}^{T} d_{k - 1} } \right| \le - \nu g_{k - 1}^{T} d_{k - 1}$ and the inductive hypothesis and combining with Eq. (39) we have:

40 $$ \frac{{g_{k}^{T} d_{k} }}{{\left\| {g_{k} } \right\|^{2} }} \ge - 1 - \frac{{\left| {g_{k}^{T} d_{k - 1} } \right|}}{{\left\| {g_{k - 1} } \right\|^{2} }} \ge - 1 + \nu \frac{{g_{k}^{T} d_{k - 1} }}{{\left\| {g_{k - 1} } \right\|^{2} }} \ge - 1 - \frac{{\nu - \nu^{k} }}{1 - \nu } = - \frac{{1 - \nu^{k} }}{1 - \nu } $$

41 $$ \frac{{g_{k}^{T} d_{k} }}{{\left\| {g_{k} } \right\|^{2} }} \le - 1 + \frac{{\left| {g_{k}^{T} d_{k - 1} } \right|}}{{\left\| {g_{k - 1} } \right\|^{2} }} \le - 1 - \nu \frac{{g_{k}^{T} d_{k - 1} }}{{\left\| {g_{k - 1} } \right\|^{2} }} \le - 1 + \frac{{\nu - \nu^{k} }}{1 - \nu } = - \frac{{1 - 2\nu + \nu^{k} }}{1 - \nu } $$

Combining the Eqs. (40) and (41), it gives $- \frac{{1 - \nu^{k} }}{1 - \nu } \le \frac{{g_{k}^{T} d_{k} }}{{\left\| {g_{k} } \right\|^{2} }} \le \frac{{1 - 2\nu + \nu^{k} }}{1 - \nu }$, when $\nu < \frac{1}{2},k \ge \frac{1}{2}$, there exists $c \triangleq \frac{1 - 2v}{{1 - v}} > 0$, such that $g_{k}^{T} d_{k} \le - c\left\| {g_{k} } \right\|^{2}$, then the search direction $d_{k}$ generated by the IPRPCG algorithm satisfies the sufficient descent condition.

The iteration step $\alpha_{k}$ is found by Wolfe's line search criterion, which satisfies:

42 $$ f\left( {x_{k} + \alpha_{k} d_{k} } \right) - f\left( {x_{k} } \right) \le \delta \alpha_{k} g_{k}^{T} d_{k} $$

43 $$ g\left( {x_{k} + \alpha_{k} d_{k} } \right)^{T} d_{k} \ge \nu g_{k}^{T} d_{k} $$

From $g_{k}^{T} d_{k} \le - c\left\| {g_{k} } \right\|^{2}$ we have:

44 $$ \left\| {d_{k} } \right\| \le c\left\| {g_{k} } \right\|,g_{k}^{T} d_{k} = - \left\| {g_{k} } \right\|^{2} $$

From Eqs. (42)–(44), we have:

45 $$ f_{k + 1} - f_{k} \le \delta \alpha_{k} g_{k}^{T} < 0 $$

46 $$ g_{k + 1}^{T} d_{k} \ge \nu g_{k}^{T} d_{k} $$

Assuming that the objective function $f\left( x \right)$ is lower bounded on the level set $\Gamma = \left\{ {x \in R^{n} \left| {f\left( x \right) \le f\left( {x_{1} } \right)} \right.} \right\}$, it is known that the sequence $\left\{ {f_{k} } \right\}$ is monotonically bounded and thus converges by combining with Eq. (45), i.e., $\mathop {\lim }\limits_{k \to \infty } f_{k + 1}$ is a constant. Assume that $f\left( x \right)$ is differentiable in some neighborhood $\Phi$ of $\Gamma$ and that $\nabla f\left( x \right)$ satisfies the Lipschitz condition, i.e., there exists $\forall x,y \in \Phi$ and a constant ${\rm Z} > 0$ such that: $\left\| {g\left( x \right) - g\left( y \right)} \right\| \le {\rm Z}\left\| {x - y} \right\|$. Combining Eq. (46) yields:

47 $$ z\alpha_{k} \left\| {d_{k} } \right\|^{2} \ge d_{k} \left( {g_{k + 1} - g_{k} } \right) \ge \left( {\nu - 1} \right)g_{k}^{T} d_{k} $$

And thus $\alpha_{k} \ge \frac{\nu - 1}{z}\frac{{g_{k}^{T} d_{k} }}{{\left\| {d_{k} } \right\|^{2} }}$. Combining this with Eq. (45) yields:

48 $$ f_{k} - f_{k + 1} \ge \delta \alpha_{k} g_{k}^{T} d_{k} \ge \frac{{\delta \left( {1 - \nu } \right)}}{z}\frac{{g_{k}^{T} d_{k} }}{{\left\| {d_{k} } \right\|^{2} }} $$

For both ends of Eq. (48) ${\text{k}} = 1,2,....,n$ are summed to give:

49 $$ f_{1} - \mathop {\lim }\limits_{k \to \infty } f_{k + 1} \ge \frac{{\delta \left( {1 - \nu } \right)}}{z}\sum\limits_{k = 1}^{\infty } {\frac{{g_{k}^{T} d_{k} }}{{\left\| {d_{k} } \right\|^{2} }}} $$

Thus there is Zoutendijk condition $\sum\limits_{k = 1}^{\infty } {\frac{{\left( {g_{k}^{T} d_{k} } \right)^{2} }}{{\left\| {d_{k} } \right\|^{2} }} < } + \infty$ holds.

Combining Eq. (44) with the Zoutendijk condition yields:

50 $$ + \infty > \sum\limits_{k = 1}^{\infty } {\frac{{\left( {g_{k}^{T} d_{k} } \right)^{2} }}{{\left\| {d_{k} } \right\|^{2} }}} = \sum\limits_{k = 1}^{\infty } {\frac{{\left\| {g_{k} } \right\|^{4} }}{{\left\| {d_{k} } \right\|^{2} }}} \ge c^{ - 2} \sum\limits_{k = 1}^{\infty } {\left\| {g_{k} } \right\|}^{2} $$

Thus the sequence $\left\{ {g_{k} } \right\}$ generated by the algorithm IPRPCG satisfies $\mathop {\lim }\limits_{k \to \infty } \left\| {g_{k} } \right\| = 0$. Then the algorithm converges globally.

## References

[1] Chen SC, Zhou HM (2016). Re-exploration to migration of seismic data. Chin. J. Geophys..

[2] Lambaré G, Virieux J, Madariaga R, Jin S (1992). Iterative asymptotic inversion in the acoustic approximation. Geophysics.

[3] Song LW, Shi Y, Liu W, Zhao Q (2022). Elastic reverse time migration for weakly illuminated structure. Applied Science..

[4] Zhou Y, Cao JX, Wang XJ, Hu JT, Wang HZ, Liu WQ (2021). An efficient illumination compensation imaging method based on reverse-time migration. Chin. J. Geophys.-Chin. Ed..

[5] Liu YZ, Liu WG, Wu Z, Yang JH (2022). Reverse time migration with an exact two-way illumination compensation. Geophysics.

[6] Vamaraju J, Vila J, Araya-Polo M, Datta D, Sidahmed M, Sen MK (2021). Minibatch least-squares reverse time migration in a deep-learning framework. Geophysics.

[7] Liu QC (2016). Improving the gradient in least-squares reverse time migration. J. Geophys. Eng..

[8] Sun XD, Ge ZH, Li ZC (2017). Conjugate gradient and cross-correlation based least-square reverse time migration and its application. Appl. Geophys..

[9] Li J, Li QC, Zhang XH (2016). Overview of progress in least squares migration studying. Prog. Geophys..

[10] Li ZC (2014). Research status and development trend of seismic offset migration imaging technology. Oil Geophys. Prospect..

[11] Chen SC, Ma ZT, Wu RS (2007). Illumination compensation for wave equation migration shadow. Chin. J. Geophys..

[12] Li C, Huang JP, Li ZC, Wang RR, Li QY (2016). Preconditioned least squares reverse time method. Oil Geophys. Prospect..

[13] Zhou HM, Chen SC, Zhou LM, Ren HR, Wu RS, Xiao GQ (2019). Reflected wave least squares reverse time migration with angle illumination compensation. Acta Geophys..

[14] Zhu F, Huang JP, Yu H (2018). Least-squares Fourier finite-difference pre-stack depth migration for VTI media. J. Geophys. Eng..

[15] Liu YS, Teng JW, Xu T, Badal J, Liu QY, Zhou B (2017). Effects of conjugate gradient methods and step-length formulas on the multiscale full waveform inversion in time domain: numerical experiments. Pure Appl. Geophys..

[16] Kim YS, Tsingas C, Jeong W (2018). Directional-oriented wavefield imaging: a new wave-based subsurface illumination imaging condition for reverse time migration. Geophys. Prospect..

[17] Yu GP, Xu T, Zhang MH, Bai ZM, Liu YS, Wu CL, Teng JW (2017). Nonlinear travel-time inversion for 3-D complex crustal velocity structure. Chin. J. Geophys..

[18] Chen SC, Wang HC (2010). Migration compensation with plane wave illumination. Chin. J. Geophys..

[19] Wu HT, Li J, Wang KP, Li X (2021). Constrained inversion of the reweighted regularized conjugate gradient method for heavy magnetic data. Comput. Tech. Geophys. Geochem. Explor..

[20] Sun JH, Zhu TY (2018). Strategies for stable attenuation compensation in reverse-time migration. Geophys. Prospect..

[21] Hu CR, Huang JQ, Liu H (2022). Multi-scale full waveform inversion of Love waves based on PRP conjugate gradients. Chin. Water Transp..

[22] Du SY, Zhang JS, Hu GM (2020). A robust data-driven AVO inversion with logarithm absolute error loss function. Acta Geophys..

[23] Du SY, Zhang JS, Hu GM (2021). Adaptive individual weight-gain AVO inversion with smooth nonconvex regularization. Acta Geophys..

[24] Du SY, Zhang JS, Hu GM (2022). Robust data-driven AVO inversion algorithm based on generalized nonconvex dictionary learning. J. Petrol. Sci. Eng..

[25] Zhang P, Liao F (2012). Research and prospect of conjugate gradient method. J. Mudanjiang Normal Univ: Nat. Sci. Ed..

[26] Yuan GL, Lu JY, Wang Z (2020). The PRP conjugate gradient algorithm with a modified WWP line search and its application in the image restoration problems. Appl. Numer. Math..

[27] Chen SC, Zhang B (2012). Steeply-dipping structures migration based on the wave field vertical and horizontal extrapolation. Chin. J. Geophys..

[28] Tang YX (2009). Target-oriented wave-equation least-squares migration/inversion with phase-encoded Hessian. Geophysics.

[29] Beydoun WB, Mendes M (1989). Elastic ray-born 12-migration/inversion. Geophys. J. Int..

[30] Zhao XL, Wu GC, Cao DP (2016). A joint sparse Bayesian inversion method for multi-scale seismic data. Oil Geophys. Prospect..

[31] Jin S, Walraven D (2003). Wave equation GSP prestack depth migration and illumination. Lead. Edge.

[32] Rong C, Jia XF (2021). An efficient local imaging strategy based on illumination analysis with deep learning. Front. Earth Sci..

